# Monitoring disease activity of ankylosing spondylitis

**DOI:** 10.2478/abm-2026-0024

**Published:** 2026-08-31

**Authors:** Editorial Office of Asian Biomedicine

**Affiliations:** Faculty of Medicine, Chulalongkorn University, Bangkok 10330, Thailand

Ankylosing spondylitis (AS) is a chronic inflammatory disease of the axial spine. The disease typically manifests as chronic back pain and progressive spinal stiffness, which affects approximately 1/200 individuals [1]. In addition to the axial spine, AS can involve sacroiliac joints, peripheral joints, digits, and entheses where tendons, ligaments, joint capsules, and fascia attach to bone. These joints and their connection points play a crucial role in the body by helping absorb mechanical shock and transmit the pulling forces of muscles, allowing normal movement and function. Therefore, inflammation of these structures can give rise to buttock pain, hip pain, peripheral arthritis, enthesitis, and dactylitis, impaired spinal mobility, and abnormal postures [1].

Axial spondyloarthritis consists of radiographic changes of the axial spine. This can lead to disabling spinal fusion and hyperkyphosis [2]. In some patients, axial inflammation occurs without definite radiographic sacroiliitis; this entity is known as non-radiographic axial spondyloarthritis [3].

Several genetic and nongenetic risk factors have been implicated in the onset of AS. Human leukocyte antigen B27 (HLA-B27) has a strong association with AS, but other genes are also involved in disease development [4]. However, most people carrying HLA-B27 do not develop AS [5]. Therefore, HLA-B27 is not a standalone screening tool.

The Bath Ankylosing Spondylitis Disease Activity Index (BASDAI) has commonly been used as a measure of symptoms and disease status in AS [6]. Platelet-to-lymphocyte ratio (PLR) can be considered as an inexpensive, dynamic marker of disease activity [7]. As inflammatory activity increases, changes in platelet and lymphocyte counts may elevate PLR, making it a potential marker [7]. In addition, serum amyloid A (SAA) is also a marker of disease activity [8].

In this issue, Yu et al. [9] reported that SAA and PLR were strongly associated with BASDAI-defined disease activity in AS, with both markers increasing across low-, moderate-, and high-activity groups. SAA showed excellent discrimination for high disease activity, and the combination of SAA and PLR improved diagnostic performance compared with either marker alone. These findings provide additional evidence supporting the potential utility of SAA and PLR as adjunctive biomarkers of disease activity in AS. These indices may serve as practical combined markers for objective assessment of disease activity during treatment, although their role should currently be considered complementary to, rather than a replacement for, established clinical and laboratory assessments. Their findings also support future investigations examining whether these biomarkers can monitor treatment response and long-term disease outcomes after acute symptoms subside. However, because the study was cross-sectional and conducted at a single center, further validation in independent and longitudinal cohorts is required before routine clinical implementation can be recommended.

An important consideration is how SAA and PLR compare with currently established disease activity measures. BASDAI remains widely used in clinical practice; however, the Ankylosing Spondylitis Disease Activity Score, which incorporates objective inflammatory markers, is increasingly favored for disease activity assessment and treatment decisions. Future studies should determine whether SAA and PLR provide incremental value beyond conventional markers such as C-reactive protein and erythrocyte sedimentation rate and whether their incorporation improves the performance of established disease activity instruments.

The practical applicability of these biomarkers also deserves consideration. PLR is inexpensive and readily available because it is derived from routine blood counts. In contrast, SAA testing may not be universally accessible and may incur additional costs in some healthcare settings. Therefore, the clinical value of combining SAA with PLR should ultimately be evaluated not only for diagnostic performance, but also for feasibility, accessibility, and cost-effectiveness.
